# Prevalence and characteristics of bladder wall thickening in dogs without lower urinary tract disease: an ultrasonographic study

**DOI:** 10.3389/fvets.2025.1713723

**Published:** 2025-12-05

**Authors:** Chloé François, Frédéric Billen, Stéphanie Noël, Anne-Laure Etienne, Laurence Seidel, Géraldine Bolen

**Affiliations:** 1 Department of Companion Animal Clinical Sciences, Faculty of Veterinary Medicine, University of Liège, Liege, Belgium; 2 University and University Hospital of Liège – Biostatistics and Research Method Center, Liege, Belgium

**Keywords:** urinary bladder, wall, anatomy, ultrasound, dog

## Abstract

**Introduction:**

Assessment of bladder wall thickness by ultrasound is influenced by bladder distension and body weight, complicating interpretation. In the author’s experience, cranial or cranio-ventral bladder wall thickening is commonly observed in dogs without lower urinary tract disease. One of our main hypotheses is that this thickening reflects physiological mucosal folding, which becomes more pronounced as bladder distension decreases. However, bladder thickening remains poorly documented in healthy dogs. This study aimed to investigate the prevalence and characteristics of bladder wall thickening in dogs without lower urinary tract disease.

**Methods:**

Medical data of 136 dogs without lower urinary tract disease signs, undergoing abdominal ultrasound and urinalysis, were retrospectively reviewed. Ultrasound data included bladder wall thickening presence and localization (cranial, ventral, cranio-ventral, …, generalized), aspect of the luminal surface (smooth-irregular) and the urine, and bladder distension (empty, mild, moderate, severe). A wall thickness ratio (maximum/minimum thickness) was calculated in cases of asymmetrical thickness. Multivariate logistic regression (*p* < 0.05) assessed associations between bladder wall thickening and other medical and ultrasound data.

**Results:**

Bladder wall thickening was observed in 42.6% of cases, predominantly in the cranio-ventral region (22.1%), followed by the cranial (11%), generalized (8%), and ventral (5%) regions. Thickening was more frequent in mildly (29.4%) and moderately (10.3%) distended bladders. Significant associations were found between thickening and age (OR = 1.16), gastrointestinal (OR = 5.49), and renal diseases (OR = 5.57). Sterilized dogs were less likely to exhibit cranio-ventral thickening (OR = 0.36). The median thickness ratio was not statistically significant across bladder sizes (*p* = 0.82): 2.0 for mildly, 1.95 for moderately, and 1.9 for severely distended bladder.

**Conclusion:**

These findings suggest that mild cranio-ventral/cranial bladder thickening is common in dogs without lower urinary tract disease with a median thickness ratio ≤ 2.0 and should not be confound with cystitis.

## 1 Introduction

Ultrasound is widely recognized as the imaging modality of choice for evaluating the urinary bladder due to its non-invasive nature, rapid image acquisition, and high diagnostic accuracy (1–3). However, assessing bladder wall thickness by ultrasound presents challenges, as it varies with both bladder distension, and animal’s weight (3). A previous study reported that mean bladder wall thickness was 2.3 mm in minimally distended bladders (0.5 mL/kg saline), 1.6 mm in mildly distended bladders (2 mL/kg saline) and 1.4 mm in moderately distended bladders (4 mL/kg saline), with a significant increase thickness observed in heavier dogs (3). In this study, the caudoventral bladder wall thickness measurement was reported as the smallest measurement, with a difference of 0.3 mm (3). Despite these findings, precise localization and characterization of bladder thickening on ultrasound remain poorly documented in healthy dogs.

Moderate bladder distension optimizes ultrasound assessment by minimizing artifactual thickening (4, 5). However, achieving optimal bladder filling during an ultrasound examination in a daily context is not always feasible. Bladder catheterization is one method used to facilitate bladder filling; although commonly rapid, this technique is associated with a risk of urinary tract infections or trauma and can be challenging, particularly in small female dogs (5, 6). Given these limitations, radiologists must carefully consider bladder filling status when interpreting bladder wall thickness.

Bladder wall thickening is a common ultrasonographic finding in dogs and is associated with pathological conditions such as infectious cystitis, urolith-associated cystitis, polypoid cystitis, or bladder neoplasia, all of which may cause focal or diffuse parietal modification (4, 5). Among these conditions, thickening of the cranio-ventral bladder wall often raises suspicion of cystitis. However, based on the authors’ clinical experience, mild cranial or cranio-ventral thickening is also frequently observed in dogs without lower urinary tract disease, even at different degrees of bladder distension. To date, no study has specifically characterized this thickening in dogs without lower urinary tract disease.

This study aims to investigate the prevalence and characteristics of bladder wall thickening in dogs without lower urinary tract disease. We hypothesized that cranial or cranio-ventral bladder wall thickening may be common in dogs without lower urinary tract disease and should be distinguished from pathological thickening. We further hypothesized that this thickening could reflect persistent mucosal foldings that become more apparent when bladder distension decrease.

## 2 Materials and methods

### 2.1 Case selection

**Figure 1:**
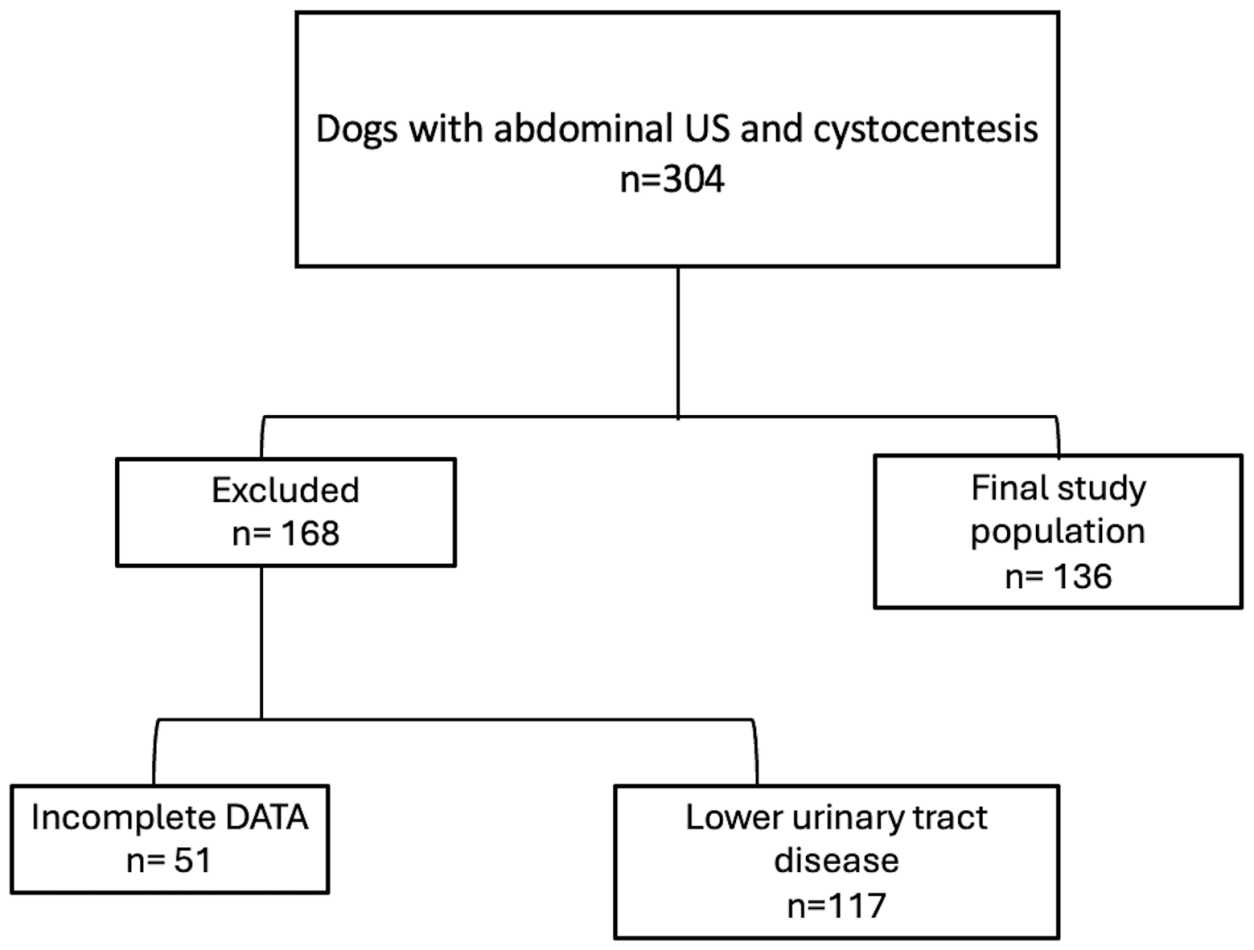
Flow diagram illustrating case selection.

The medical records of client-owned dogs referred to the Liège University Veterinary Clinic between April 2022 and September 2023 were retrospectively reviewed. Inclusion criteria required dogs that have undergone both abdominal ultrasound and urinalysis, with urine samples collected via cystocentesis. Exclusion criteria included a history of clinical signs related to the lower urinary tract (e.g., pollakiuria, hematuria, dysuria, stranguria, or incontinence), as well as diagnoses of lower urinary tract disease such as cystitis, urolithiasis, bladder polyps or neoplasia. Dogs displaying gravitationally dependent sediment, compatible with vesical sand on ultrasound were also excluded. A total of 304 dogs that had undergone both abdominal ultrasonography and cystocentesis met the initial screening criteria. Of these, 168 were excluded, including 117 due to lower urinary tract disease and 51 due to incomplete ultrasonographic or urinalysis data. The final study population therefore consisted of 136 dogs without lower urinary tract disease that had undergone both abdominal ultrasonography and cystocentesis (Figure 1).

### 2.2 Medical record

Data were extracted from electronic medical records and included signalment (breed, neutered status, sex, age at presentation, and weight), final diagnosis, co-morbidities (e.g., cystitis-promoting diseases such as endocrinopathies, upper urinary tract infections, reproductive tract infections, and immunosuppression), treatments administered prior to ultrasound, and urinalysis results, including sediment analysis and urine culture results.

Sediment analysis was used to record the presence of bacteriuria. Pyuria was defined as >5 white blood cells (WBCs) per high-power field (hpf). When urine culture results were available, urinary tract infection was diagnosed based on the isolation of >1 × 10^3^ colony-forming units (CFU)/mL from samples obtained by cystocentesis (7). Crystalluria was also assessed during sediment analysis.

### 2.3 Ultrasound images review

Ultrasound images were acquired by various operators, including board-certified radiologists, radiology assistants, and radiology residents, with dogs positioned in dorsal recumbency. Imaging was performed using either a Canon Aplio Alpha 450 system (Europe, Zoetermeer, the Netherlands) with a range of transducers [microconvex 11MC4 (4–11 MHz), linear 18 L7 (4.5–18 MHz), linear 17LH7 (4.5–17 MHz), and linear 14 L5 (3.8–10 MHz)] or a Hitachi Arietta 850 SE system (Tokyo, Japan) with linear (5–18 MHz) and microconvex (4–10 MHz and 4–8 MHz) transducers. Ultrasound probe and frequency were adapted according to the dog’s conformation as in clinical daily use.

All ultrasonographic images of the urinary bladder from dogs without lower urinary tract disease were retrospectively reviewed using an image-viewing program (Impax version 8.1.2, Agfa HealthCare N, Mortsel, Belgium) by a second-year European College of Veterinary Diagnostic Imaging (ECVDI) resident under the supervision of an ECVDI board-certified radiologist. Cases with uninterpretable or missing bladder images were excluded.

**Figure 2:**
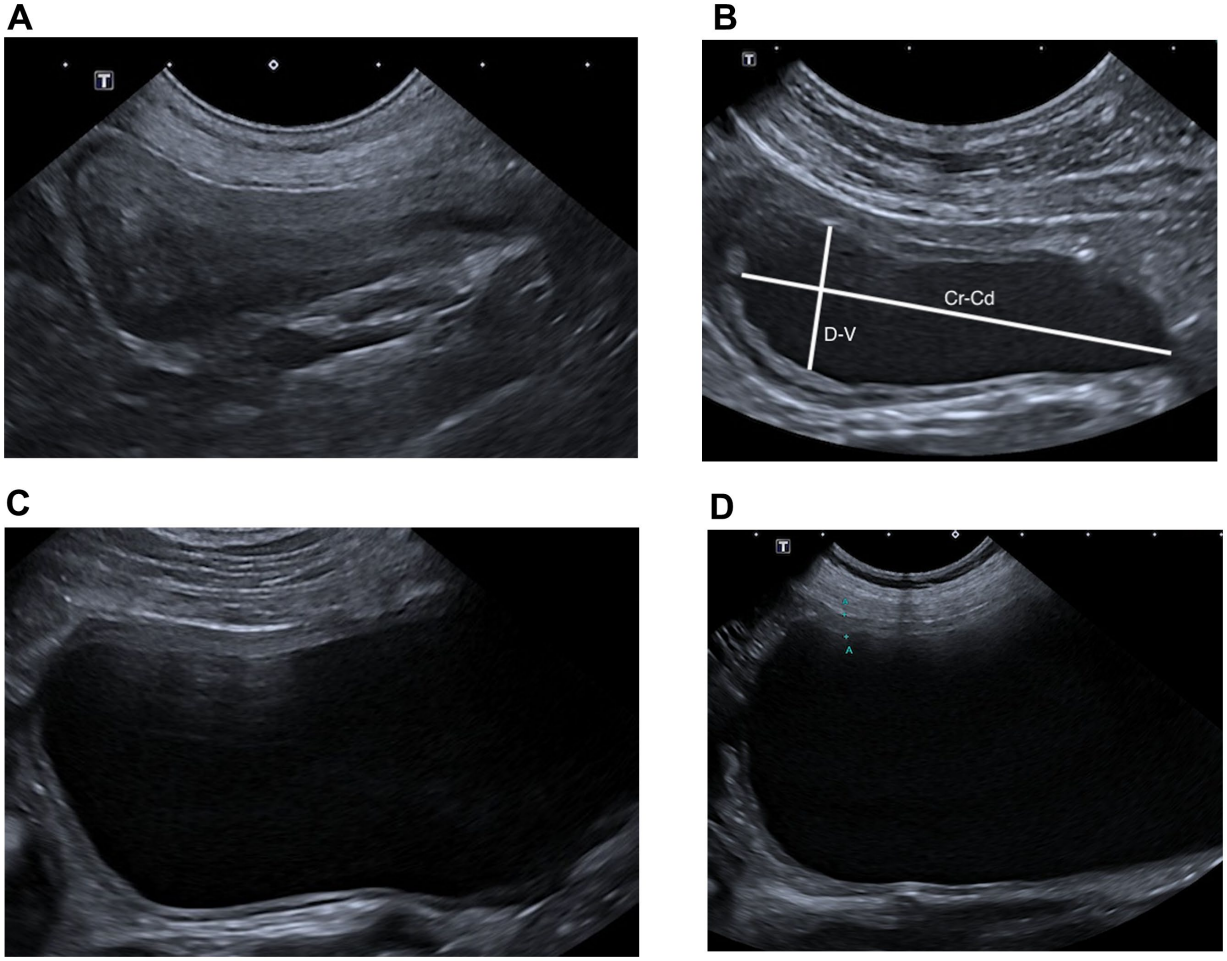
Subjective classification of bladder distension. Sagittal ultrasonographic images illustrating four degrees of bladder distension: **(A)** empty bladder, **(B)** mildly distended bladder, **(C)** moderately distended bladder, and **(D)** severely distended bladder. In panel B, the white lines indicate the cranio-caudal (Cr–Cd) and dorso-ventral (D–V) axes used for bladder area estimation.

Bladder distension was subjectively classified as empty, mildly, moderately, or severely distended (Figure 2). Bladder area was estimated in the sagittal plane by multiplying the cranio-caudal (Cr-Cd) axis by the dorso-ventral (D-V) axis (Figure 2), using the formula: estimated area (cm^2^) = Cr-Cd length (cm) × D-V length (cm) and this value was then normalized to body weight (kg) (Area/weight). Ultrasound assessments included evaluation of bladder wall characteristics, specifically the presence (yes/no) and localization of wall thickening (cranio-ventral, cranio-dorsal, cranial, caudo-ventral, caudo-dorsal, caudal, ventral, dorsal, or generalized), as well as the appearance of the parietal luminal surface (smooth or irregular). Bladder wall thickness was measured retrospectively as the distance from the outer serosa to the inner mucosa (8). For each case, the maximum bladder wall thickness was measured. Because bladder distension varied among cases, wall thickness was interpreted according to established reference values: thickening was defined as a measurement exceeding 2.3 mm in minimally distended bladders, 1.6 mm in mildly distended bladders, and 1.4 mm in moderately to severely distended bladders (3). In cases of asymmetric thickening, a difference greater than 0.3 mm between the thickened and non-thickened wall was considered significant (3). To further characterize asymmetry, a ratio was calculated between the maximum and minimum bladder wall thickness. Additionally, the ultrasonographic aspect of the urinary contents (anechoic, echoic/hyperechoic particles, echoic cluster) was documented.

### 2.4 Statistical analysis

Variables were summarized as mean ± standard deviation (SD) or median with interquartile range (Q1–Q3) for quantitative variables and as frequencies and percentages for qualitative variables. The normality of quantitative data was assessed using the Shapiro–Wilk test, and logarithmic transformation was applied when appropriate. All parameters were compared with respect to bladder distension (mild, moderate, severe) by an ANOVA-1 or Kruskal-Wallis test for quantitative parameters and by a chi-square test for qualitative parameters. The presence of bladder wall thickening was analyzed using logistic regression. Odds ratios (OR) were reported. The same analysis was repeated on cranial, cranio-ventral thickening but only relevant results were reported. Multivariate logistic regression was performed using stepwise selection (entry threshold: *p* < 0.10). Association between maximum bladder wall thickness measurement and weight was assessed by the Pearson’s correlation. Statistical significance was defined as *p* < 0.05. All analyses were performed using SAS software (version 9.4; SAS Institute Inc., Cary, NC, United States).

## 3 Results

### 3.1 Clinicopathology findings

As mentioned above, a total of 136 dogs without lower urinary tract disease met all inclusion criteria and constituted the final study population. This cohort represented 44 breeds (Table 1). Age (years), weight (kg), and sex distributions according to bladder distention are detailed in Tables 2. Abdominal ultrasound examination was performed to investigate gastrointestinal disorders (23.5%, *n* = 32), renal disorders (19.1%, *n* = 26), lymphoid and hematopoietic disorders (15.4%, *n* = 21), endocrinopathies (13.2%, *n* = 18), hepatobiliary or pancreatic disorders (9.6%, *n* = 13), orthopedic and neurologic disorders (3.7%, *n* = 5), reproductive disorders (2.9%, *n* = 4), respiratory disorders (2.9%, *n* = 4), and for other reasons (9.6%, *n* = 13).

**Table 1:** Breed distribution.

Breed	N
American Staffordshire Terrier	1
Australian Shepherd	5
Beagle	3
German Shepherd	3
Croatian Shepherd	1
Belgian Shepherd	6
Romanian Raven Shepherd	1
Swedish Vallhund	1
Maltese	7
Border Collie	6
French Bulldog	3
Bernese Mountain dog	4
Ardennes Cattle Dog	1
Boxer	1
German Shorthaired Pointer	1
Braque d’Auvergne	1
Weimaraner	2
Toy Poodle	1
Pug	1
Chihuahua	8
Cavalier King Charles	4
English Cocker Spaniel	5
Dalmatian	1
Papillon	1
Eurasian	1
Flat-Coated Retriever	2
Finnish Lapphund	1
Fox-Terrier	1
Golden Retriever	3
Belgian Griffon	1
Siberian Husky	2
Jack Russell Terrier	3
Labrador Retriever	7
Leonberg	1
Podenco	1
Rottweiler	1
Shar Pei	2
Shih Tzu	4
Shiba Inu	1
Spitz	3
English Springer Spaniel	1
Dachshund	3
West Highland White Terrier	4
Yorkshire	4
Cross Breeds	22

**Table 2:** Age (years), weight (kg), and sex distributions according to bladder distention.

Variable	Empty (*n* = 2)	Mild (*n* = 45)	Moderate (*n* = 55)	Severe (*n* = 34)	*p*-value (without empty)
	Mean ± SD Median [Q1–Q3] Number (%)	Mean ± SD Median [Q1–Q3] Number (%)	Mean ± SD Median [Q1–Q3] Number (%)	Mean ± SD Median [Q1–Q3] Number (%)	
Age (years)	14.0 ± 0.0	7.80 ± 4.14	6.15 ± 4.39	8.33 ± 4.17	0.039
Weight (kg)	17.6 [13.0–22.2]	17.0 [10.2–27.0]	12.4 [7.00–27.0]	13.3 [6.00–22.7]	0.18
Weight range					0.063
<10 Kg	0 (0.0)	11 (24.4)	21 (38.2)	16 (47.1)	
10–30 Kg	2 (100.0)	29 (64.4)	23 (41.8)	16 (47.1)	
>30 Kg	0 (0.0)	5 (11.1)	11 (20.0)	2 (5.9)	
Sex					0.95
Entire male	0 (0.0)	6 (13.3)	9 (16.4)	3 (8.8)	
Castrated male	0 (0.0)	16 (35.6)	18 (32.7)	14 (41.2)	
Entire female	0 (0.0)	5 (11.1)	7 (12.7)	5 (14.7)	
Spayed female	2 (100.0)	18 (40.0)	21 (38.2)	12 (35.3)	

Reported comorbidities included hyperadrenocorticism (13.2%, *n* = 18), diabetes mellitus (5.1%, *n* = 7), reproductive tract infection (1.4%, *n* = 2) and upper urinary tract infection (0.7%, *n* = 1). At the time of ultrasound examination, 11 dogs (8.1%) were receiving antibiotics, 9 (6.6%) were on corticosteroid therapy, and 4 (2.9%) were treated with nonsteroidal anti-inflammatory drugs.

Urinalysis revealed a median pH of 6.5 (range: 5–9). While most values fell within the expected physiological range for dogs (5.0–7.5) (9), a small number (*n* = 3) of isolated alkaline values (up to pH 9) were recorded.

Sediment analysis revealed crystals in 8 dogs (5.9%), bacteria in 6 dogs (4.4%), bacteria and WBCs in 2 dogs (1.5%), and WBCs in one dog (0.7%).

Urine culture results were available for six dogs. Three cultures were positive: one dog with both bacteria and WBCs on sediment was positive for *Pseudomonas aeruginosa* (20,000 CFU/mL) and *Citrobacter braakii* (10,000 CFU/mL), and two dogs with bacteria only on sediment were positive for *Escherichia coli* (>100,000 CFU/mL and >30,000 CFU/mL). The remaining three cultures were negative: one had a normal sediment, one had bacteria only in the sediment, and one had WBCs only in the sediment.

### 3.2 Bladder ultrasound findings

Bladder distention was subjectively assessed in 136 dogs, classified as empty (2/136; 1.5%) (Figure 2A), mildly (45/136; 33.1%) (Figure 2B), moderately (55/136; 40.4%) (Figure 2C), or severely (34/136; 25%) distended (Figure 2D). Table 3 shows a comparison of ultrasound parameters as a function of bladder distension. The median bladder area for mildly, moderately, and severely distended bladders was 8.4 cm^2^ (Q1–Q3: 5.4–12.7), 17 cm^2^ (Q1–Q3: 9.6–26.6), and 33.3 cm^2^ (Q1–Q3: 22.8–51.9), respectively and was statistically different across bladder distension categories (*p* < 0.0001). The median relative bladder area per body weight was 0.54 cm^2^/kg (Q1–Q3: 0.39–0.68) for mildly distended bladders, 1.10 cm^2^/kg (Q1–Q3: 0.81–1.86) for moderately distended bladders, and 2.83 cm^2^/kg (Q1–Q3: 2.30–4.07) for severely distended bladders (*p* < 0.0001).

**Table 3:** Comparison of ultrasound parameters as a function of bladder distension.

Variable	Absent (*n* = 2)	Mild (*n* = 45)	Moderate (*n* = 55)	Severe (*n* = 34)	*p*-value (without empty)
	Median [Q1–Q3] Number (%)	Median [Q1–Q3] Number (%)	Median [Q1–Q3] Number (%)	Median [Q1–Q3] Number (%)	
Thickening					<0.0001
Absent	0 (0.0)	5 (11.1)	41 (74.5)	32 (94.1)	
Present	2 (100.0)	40 (88.9)	14 (25.5)	2 (5.9)	
Localization of the thickening					<0.0001
Cranio-ventral	0 (0.0)	18 (40.0)	10 (18.2)	2 (5.9)	
Ventral	0 (0.0)	4 (8.9)	1 (1.8)	0 (0.0)	
Cranial	0 (0.0)	12 (26.7)	3 (5.5)	0 (0.0)	
Generalized	2 (100.0)	6 (13.3)	0 (0.0)	0 (0.0)	
Maximum bladder wall (mm)	5.25 [5.00–5.50]	3.30 [2.50–4.30]	1.20 [0.90–1.80]	1.10 [0.90–1.40]	<0.0001
Ratio (max/min)	/	2.00 [1.70–2.50]	1.95 [1.80–2.80]	1.90 [1.80–2.00]	0.82
Parietal surface					
Smooth	1 (50.0)	27 (60.0)	50 (90.9)	34 (100.0)	<0.0001
Irregular	1 (50.0)	18 (40.0)	5 (9.1)	0 (0.0)	
Bladder area (cm^2^)	0.00 [0.00–0.00]	8.40 [5.40–12.7]	17.0 [9.60–26.6]	33.3 [22.8–51.9]	<0.0001
Relative bladder area (cm^2^/kg)	0.00 [0.00–0.00]	0.54 [0.39–0.68]	1.10 [0.81–1.86]	2.83 [2.30–4.07]	<0.0001

**Figure 3:**
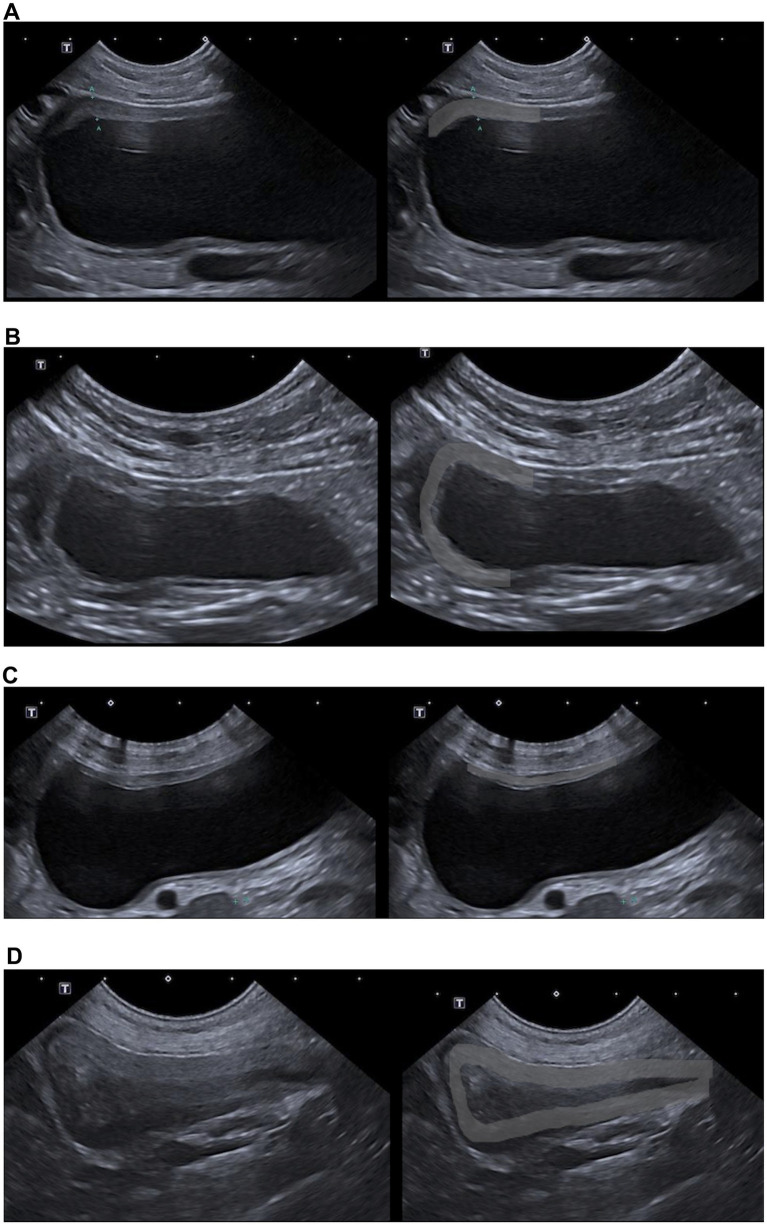
Localization of bladder wall thickening. Sagittal ultrasonographic images showing different patterns of bladder wall thickening: **(A)** cranio-ventral thickening, **(B)** cranial thickening, **(C)** ventral thickening, and **(D)** generalized bladder wall thickening. For each pattern, the image on the right duplicates the corresponding view and highlights the thickened region with a light-gray overlay for improved visualization.

Bladder wall thickening was observed in 58 of the 136 dogs without lower urinary tract disease (42.6%), regardless of location. Thickening was localized to the cranioventral wall in 30 dogs (22.1%), the cranial wall in 15 dogs (11%), and the ventral wall in 5 dogs (3.6%), while 8 dogs (5.9%) exhibited generalized thickening. Examples of each localization pattern are shown in Figure 3 (cranioventral: Figure 3A; cranial: Figure 3B; ventral: Figure 3C; generalized: Figure 3D).

Cranioventral bladder wall thickening (Figure 3A) was the most common pattern observed across all bladder distension types (*p* < 0.001). Although prevalent, its occurrence decreased as bladder distension increased, being significantly less frequent in moderately (OR = 0.319) and severely distended bladders (OR = 0.093) compared to empty or mildly distended ones.

Maximum bladder wall thickness significantly differed according to bladder distension (*p* < 0.0001). Median thickness was 3.3 mm (Q1–Q3: 2.5–4.3) in mildly distended bladders, 1.2 mm (Q1–Q3: 0.9–1.8) in moderately distended bladders, and 1.1 mm (Q1–Q3: 0.9–1.4) in severely distended bladders. The median ratio between the maximum and minimum bladder wall thickness was not statistically different across bladder size categories: 2.0 for mild (Q1–Q3: 1.7–2.5), 1.95 for moderately (Q1–Q3: 1.8–2.8), and 1.9 for severely (Q1–Q3: 1.8–2.0) distended bladders (*p* = 0.82).

Among cases with thickening, the luminal surface appeared smooth in 35/58 (60.3%) and irregular in 23/58 (39.7%). The luminal parietal surface was more frequently smooth in moderately and severely distended bladder than in mildly distended bladder (*p* < 0.0001).

Multivariate analysis revealed that bladder wall thickening increased with age (OR = 1,16, *p* = 0.027) and was more frequent in dogs investigated for gastrointestinal (OR = 5.49, *p* = 0.012) or renal (OR = 5.57, *p* = 0.018) diseases. An irregular luminal surface of the bladder wall was frequently associated with wall thickening (OR = 11.6, *p* = 0.018). Cranioventral wall thickening was less frequent in sterilized dogs (OR = 0.36, *p* = 0.032). Furthermore, maximum bladder wall thickness was positively correlated with body weight (*r* = 0.3, *p* = 0.0005).

Among the six dogs with bacteria detected on sediment analysis, three showed thickening localized to the cranial (*n* = 2) or cranioventral (*n* = 1) bladder wall, with moderately (*n* = 2) and severely (*n* = 1) distended bladders. The three others exhibited no bladder wall thickening with moderately (*n* = 1) and severely distended bladders (*n* = 2). The dog with WBCs alone in the sediment demonstrated no thickening. Among the two dogs with both WBCs and bacteria in the urine sediment, one exhibited no thickening with a severely distended bladder, whereas the other presented with generalized thickening in an empty bladder.

## 4 Discussion

This study provides new insights into bladder wall thickening detected by ultrasound in dogs without lower urinary tract disease. Our findings indicate that bladder wall thickening is a common observation, occurring in 42.6% of cases (*n* = 58/136).

As previously described (3), bladder wall thickness is influenced by the degree of bladder distension with mean bladder wall thickness being inversely correlated to bladder distension. Our findings are consistent with this observation, as bladder wall thickening was significantly more frequent in mildly (29.4%) and moderately (10.3%) distended bladders, and rare in severely distended ones (1.4%). Furthermore, our study revealed that the distribution of thickening was also affected by the degree of distension, with cranioventral and cranial thickening more commonly observed in mildly distended bladders. This regional variation in thickening is noteworthy, as it contrasts with human medicine, where bladder wall thickness is generally reported to vary uniformly across all regions in response to changes in bladder volume (10, 11).

Cranioventral thickening is often associated with cystitis (4, 5, 8), although it is important to note that a normal bladder wall appearance can still be observed in some cases of cystitis. In our study, the relatively high prevalence of cranioventral thickening in dogs without lower urinary tract disease (22.1%, *n* = 30/136) suggests that mild cranio-ventral thickening alone should not immediately be attributed to cystitis in the absence of clinical signs or supporting laboratory findings. Furthermore, we calculated a median ratio between maximum and minimum parietal thickness of 1.9 to 2, regardless of bladder size. This ratio may therefore still be considered non-pathological in dogs with asymmetric bladder thickness without associated symptoms.

In our study, the luminal surface of the bladder wall appeared smooth more frequently in moderately and severely distended bladders than in mildly distended ones. This finding is consistent with previous reports (3) and is likely explained by physiological mucosal folding that occurs when the bladder is underfilled. In mildly distended bladders, these normal folds can result in an irregular appearance of the luminal surface on ultrasound. Consequently, an irregular mucosal surface should not be immediately interpreted as indicative of cystitis without taking the degree of bladder distension into account.

Distinguishing pathological from non-pathological bladder wall thickening solely by ultrasound can be challenging. However, our findings suggest that mild cranioventral thickening with an irregular luminal surface in underfilled bladders often reflects physiological mucosal folding, which typically decreases once moderate or severe distension is achieved. A moderate degree of bladder filling is therefore advised when evaluating the bladder wall (4, 5), as persistent thickening despite adequate distension is more suggestive of pathology. In asymptomatic dogs, a thickness ratio ≤2.0 may also support a non-pathological interpretation. Due to the retrospective nature of this study, additional indicators such as regional steatitis or lymphadenopathy could not be assessed and could be explored in future prospective studies to help differentiate pathological from non-pathological thickening.

Mean maximum bladder wall thickness was 3.3 mm in mildly distended bladders, 1.2 mm in moderately distended bladders, and 1.1 mm in severely distended bladders. Notably, the mean thickness observed in mildly distended bladders exceeded the reference value of 1.6 mm cited in the Materials and methods section. This reference was derived from a prospective study in which bladder filling was standardized using a saline infusion of 2 mL/kg in the mildly distended group. In our study, due to its retrospective design, precise evaluation and standardization of bladder volume were more challenging. This lack of control over bladder distension may explain the observed discrepancies in wall thickness, particularly in mildly filled bladders where physiological mucosal folds can mimic or overestimate thickening.

Bladder wall thickening was more common in older dogs. This finding is consistent with observations in human medicine, where age-related bladder thickening has been attributed to muscular hypertrophy, fibrosis, and increased interstitial collagen deposition (9, 10). In dogs, cranio-ventral thickening could also be the result of previous inflammatory processes, such as episodes of cystitis. Although dogs with a history of lower urinary tract symptoms were excluded from the study, it cannot be ruled out that undiagnosed or asymptomatic episodes may have contributed to this structural change.

Bladder wall thickening was more frequently observed in dogs with gastrointestinal or renal disorders in the present study. Although the underlying mechanisms remain unclear, systemic diseases may influence the lower urinary tract. In dogs, chronic kidney disease has been associated with a notable prevalence of subclinical bacteriuria and bacterial cystitis (18.1% of cases, *n* = 33/182), suggesting that renal pathology may predispose to urinary tract alterations even without clinical signs; however, the underlying mechanism was not elucidated in that study (12). Additionally, recent veterinary evidence supports a gut–urinary axis: dietary characteristics and gastrointestinal microbiome composition can modulate the urinary microbiome in healthy dogs (13), indicating that gastrointestinal disease may indirectly influence bladder physiology. In human medicine, links between interstitial cystitis and inflammatory bowel disease, as well as between bladder dysfunction and chronic kidney disease, have been described, highlighting shared inflammatory and neurogenic pathways (14, 15). Further research is needed to clarify the clinical relevance of bladder wall thickening in dogs with systemic disorders.

Our study found that sterilized dogs had a lower prevalence of cranioventral bladder wall thickening compared to intact dogs, suggesting a potential hormonal influence on urothelial structure. The influence of sex steroid hormones, such as estrogen and testosterone, on urinary tract morphology is well-documented due to the common embryonic origin of the urinary and genital systems (16). Research by Yu et al. demonstrated that ovariectomized rats had thinner bladder walls and increased collagen deposition, while estrogen reversed these changes, and testosterone further increased bladder wall thickness (16). These findings suggest that sex hormones may influence regional bladder wall morphology, supporting the interpretation of cranioventral thickening as an incidental physiological variant which may be affected by sterilization. However, further investigation in canine populations is warranted, as this hormone-related effect has not been previously documented in dogs (3).

Among the 136 dogs studied, 8 had bacteria detected in urine sediment despite the absence of lower urinary tract symptoms. This suggests the presence of subclinical bacteriuria, though confirmatory urine cultures were not performed in all cases. Subclinical bacteriuria is defined as the presence of bacteria in urine, confirmed via culture, in the absence of clinical signs of urinary tract infection (17). Reported prevalence in healthy dogs ranges from 2.1 to 12% (17) and our results were in this range. Interestingly, half of the dogs with subclinical bacteriuria in our study also exhibited bladder wall thickening. This observation raises the possibility that subclinical bacteriuria may be associated with bladder wall thickening. However, this finding should be interpreted with caution due to the small sample size of dogs with subclinical bacteriuria.

The main limitation of this study is its retrospective design. Bladder imaging was not standardized, which may have affected measurement accuracy, but this reflects real clinical practice. Additionally, bladder distension was estimated subjectively, and bladder area was assessed using two-dimensional sagittal plane measurements, which do not fully account for three-dimensional bladder volume. Nevertheless, the estimated area showed statistically significant differences across subjectively defined distension categories. Although several validated formulas exist in veterinary medicine for bladder volume estimation (6), their application was not feasible due to the absence of transverse plane images for all patients in this study. As previously mentioned, bladder distension influences wall thickness. However, the wall thickness ratio (maximum/minimum thickness), which remained consistent across different levels of bladder distension, helped reduce this variability. Another important limitation is the absence of systematic urine culture. Only a small subset of dogs underwent culture, which prevents full assessment of the prevalence of subclinical bacteriuria. This is particularly relevant as some dogs exhibited bacteriuria or pyuria on sediment analysis. Therefore, the possibility that a proportion of dogs had undiagnosed subclinical urinary tract infection cannot be excluded. Additionally, few dogs were on antibiotics or corticosteroids at the time of sampling. Both medications may suppress bacteriuria or pyuria, potentially masking signs of lower urinary tract infection and underestimating its prevalence. This therapeutic effect must therefore be considered as a potential confounder in interpreting urinalysis. Finally, despite the large study population, potential selection bias cannot be ruled out, as only dogs that underwent both ultrasound and urinalysis for pre-existing conditions were included.

Future prospective studies incorporating standardized bladder filling protocols, and additional diagnostic modalities such as histopathology or advanced imaging would help refine our understanding of non-pathologic bladder wall thickening in dogs.

## 5 Conclusion

Bladder wall thickening, particularly in the cranio-ventral/cranial region, is a common and often incidental finding in dogs without clinical signs of lower urinary tract disease, likely representing persistent physiological mucosal foldings. The prevalence of these thickening decreases as bladder distension increases, further emphasizing the impact of bladder filling on wall thickness measurements. Given these observations, clinicians should exercise caution when interpreting bladder thickening, particularly in the absence of lower urinary symptoms and if the ratio between the maximum and minimum bladder wall thickness is ≤2.0.

## Data Availability

The raw data supporting the conclusions of this article will be made available by the authors, without undue reservation.
